# Ways to Make Medical Students Competent Professionally: View of Medical Students

**DOI:** 10.31729/jnma.7035

**Published:** 2021-12-31

**Authors:** Manoj Khadka, Bibash Kunwar

**Affiliations:** 1 Nepalese Army Institute of Health Sciences, Sanobharyang, Kathmandu, Nepal

**Keywords:** *competency-based education*, *medical ethics*, *medical students*

## Abstract

Medical students in Nepal learn passively by gaining encyclopedic knowledge, with little focus on the application of that knowledge to clinical scenarios and other soft skills like communication. This raises the question that whether medical students will be competent enough to serve their society in the future or not. The article highlights the domains where medical students should focus apart from medical knowledge to be competent enough to meet the health needs of society.

## INTRODUCTION

As a medical student, have you ever been asked for medical suggestions from friends, parents, relatives, or neighbors? It's hard to escape from these situations if you are a medical student, isn't it? So, if you answered yes to the previous questions, we have a few more for you to answer honestly. Were you able to solve their queries? Were they as well you satisfied with your answers?

Medical students often face health-related questions not only during their exams but also in real life where the questions are fired from their near and dear ones. We, as medical students, prepare for the exam viva but are we prepared for the viva taken by our friends, family, and society in real life? Does our medical education make us competent enough to pass the real-life viva too?

Also, are we as medical students well trained for effective communication skills? Patients believe that some kind words from doctors can heal half of their suffering. Good communication skills can have a paramount influence on patients' health because, with such skills, doctors can acquire relevant information needed for diagnosis, encourage them to follow the advice, and adhere to treatment.^1^ Likewise, another important dimension of clinical practice, medical ethics, is limited to theories in medical school, leaving us doubtful of our ability to work as ethically competent practitioners in the future.

These are just a few of many things that haunt medical students. But what can be done to make medical students competent enough? There are various ways to address this challenge and here we present a few of them.

## COMPETENCY-BASED MEDICAL EDUCATION (CBME)

The teaching-learning activities that we currently have, focus more on acquiring knowledge passively rather than the application of such knowledge in clinical situations. The assessment methods also give more priority to gaining knowledge the day before the exam and vomiting on the exam day instead of assessing students' attitudes and skills, be it clinical or soft skills associated with the communication.

Then how does CMBE helps in making the medical students competent enough? In CBME, students play an active role where teaching-learning and assessment method focuses on making medical graduates competent enough to satisfy the health needs in the society.^2^ Here, students are assessed based on how they apply their knowledge to clinical situations.^3^ It emphasizes that in addition to good medical knowledge, medical students should be competent in other essential domains like communication too.

There are various competency frameworks. One of them is the CanMEDS framework which defines the competency of a physician in terms of these seven roles: medical expert, communicator, collaborator, manager, health advocate, scholar, and professional.^4^ In a prospective follow-up study among Swedish medical students, respondents considered the roles of a medical expert, scholar, and communicator as the most important.^4^ However other roles should also be given equal priority. CanMEDS emphasizes communication and collaboration with patients, peers, and other staff, which have equal value as medical education in students' life.

Another framework from the Accreditation Council for Graduate Medical Education (ACGME) endorses six domains of core competency: patient care, medical knowledge, professionalism, interpersonal and communication skills, practice-based learning and improvement, and systems-based practice.^3,5^ If we see different frameworks for competency, they just don't focus on medical knowledge but also on other aspects required for becoming a competent physician. So, the CBME has the potential to bring a paradigm shift in medical education if incorporated in undergraduate medical education programs as medical students will have a good command of medical knowledge, clinical skills, communication skills, and other essential domains in professional life.

## CLINICAL ETHICAL COMPETENCE

Clinical ethical competence is more than just knowing theories, concepts, and principles related to ethics (cognitive part). It also involves the ability to apply the knowledge (application part) and actual performance in a real scenario (performance part). This can be demonstrated in terms of a pyramid where the cognitive part lies at the bottom, application in the middle, and performance at the top. It can also be simply interpreted as a Know-Can-Do model, where students show the knowledge part (know), perform in an observed setting in real or simulated patients (can), and do in an uncontrolled and unobserved clinical setting (do).^6^

The basic medical ethics curriculum in Nepal focuses more on the bottom part of the pyramid (cognitive part) or the Know part, but for medical students to be ethically competent, they should climb higher steps of the pyramid too. Though assessing the 'Do part' can be a bit challenging at the undergraduate level, the former 'Know' and 'Can' part needs to be assessed among medical students to make them ethically competent.

## PERSONAL HEALTH AND WELL-BEING

While struggling to be competent enough and meet the standards of the noble medical profession, medical students often neglect their health and wellbeing. Those scary nights before exams and viva are some of the culprits which add misery to the medical students' life by disturbing their sleep, food, and bowel habits. There is evidence from the literature that medical students give little time to self-care and well-being.^7^ A cross-sectional study among medical students and residents of a medical school in Nepal showed a high prevalence of burnouts (37.6%), anxiety (46.3%), and depression (29.1%) among medical students, and the major stressor being academic-related factors.^8^ This demands a student-friendly curriculum that promotes both the learning and wellbeing of students. The well-being of medical students has no other substitute and deserves uniform attention as other domains to be competent physicians in society.

## WAYS FORWARD

Medical students at the time of graduation should be brilliant not only in medical knowledge but in other aspects too like effective communication and medical ethics. Only a competent doctor is capable of fulfilling the health needs of society. And the journey to becoming a competent doctor begins with the training of medical students proficiently, encompassing the values of competency-based medical education (CBME), clinical ethical competence, and caring for students' health and wellbeing.
